# Analysis and comparison of monofocal, extended depth of focus and trifocal intraocular lens profiles

**DOI:** 10.1038/s41598-022-12694-4

**Published:** 2022-05-23

**Authors:** Juan J. Miret, Vicente J. Camps, Celia García, Maria T. Caballero, Juan M. Gonzalez-Leal

**Affiliations:** 1 Group of Optics and Visual Perception, Department of Optics, Pharmacology and Anatomy, University of Alicante, Crta San Vicente del Raspeig s/n 03016, San Vicente del Raspeig, Alicante, Spain; 2 Department of Condensed Matter Physics, Faculty of Sciences, University of Cadiz, Cadiz, Spain

**Keywords:** Biophysics, Biotechnology, Optics and photonics

## Abstract

To test the feasibility of using profilometers to extract information about IOL surfaces design. A standard monofocal IOL (Tecnis 1), a monofocal IOL that provided some depth of focus (Eyhance), an extended depth of focus IOL based on refractive optics (Mini Well) and a trifocal IOL based on diffractive optics were used in this study (Tecnis Synergy). The surface topography of the IOLs was measured by using a multimode optical profilometer. Posterior surface of Tecnis 1 IOL was spherical and the anterior surface aspherical. In the Eyhance IOL, posterior surface was spherical and anterior surface did not fit to any of our reference surfaces, indicating a higher order aspheric surface design. In the Mini Well Ready IOL, a best-fit sphere surface was obtained for the second surface and a high order aspherical surface design was deduced for the first surface. The anterior surface of the Synergy IOL was aspherical and the base curve of the diffractive structure fitted very well to a spherical surface. To consider an aspheric surface as possible best-fit surface provided more information than if only best-fit spherical surface was considered. The high order aspheric surface designs employed in the IOLs studied presented differences, regarding best-fit asphere surface, higher than 1 micron. These differences were correlated with the generation of spherical aberration complex profiles (with Zernike terms higher than 4th order) and with the production of distinct amounts of depth of focus. This method was also useful to deduce the base curve of diffractive surfaces.

## Introduction

Cataract removal through an intraocular lens is one of the most prolific fields of research and development in ophthalmology as evidenced by the large number of new intraocular lenses that have appeared in recent years. These advances are mainly motivated by the need to provide patients not only with good visual quality for far distances but also for near and intermediate distances. This is the reason why the emergence of new multifocal intraocular lens designs has taken over most of intraocular lens innovations. Multifocal intraocular lenses (MIOLs) can be made using refractive, diffractive surfaces or combinations of both. MIOLs can be called bifocal o trifocal lenses depending on they provide two or three foci, respectively^1^. Multifocal refractive lenses use different refractive zones in the same surface that produce multiple powers. Diffractive multifocal lenses have a multiscaled surface (named Fresnel surfaces or zones) that rises in concentric rings from the edge to the center. The design of these MIOLs is based in Fourier optics whose theoretical basis has already been extensively explained^1–3^. However, the development of the Extended Depth of Focus IOLs (EDOF IOLs) has been one of the latest breakthroughs in intraocular lens design. The basic principle behind these lenses is to extend the range of focus above a defined functional visual acuity threshold to provide useful distance and intermediate vision with monotonically decreasing visual acuity from the best distance focal point^4^. New high order aspheric surface designs have been developed to obtain this focus elongation^1,2^. All these advances in lens design have increased the complexity of lens surface fabrication. It is in this context when an objective quality control of lens manufacturing takes on enormous importance. In most cases this control has been done by designing optical setups to determine parameters related to the optical quality of the IOL. Parameters as the as Modular Transfer Function (MTF), Point Spread Function (PSF) or Strehl ratio have been used for this purpose^2,5–8^. A combination of optical bench measurements and simulations was developed by Camps et al. in order to obtain the same parameters but with the advantage of using real data patients in these simulations^5,6,9^. This new method based on the wavefront of the IOL allowed to know the light propagation through different surfaces. As consequence, simulations of some optical parameters (as MTF or PSF) and simulation vision optotypes were obtained. A less explored possibility to control the optical quality of the IOLs is to measure the profile of both surfaces. Some authors that studied or proposed new IOLs designs used this method to experimentally check the IOL profile^2,3,10,11^. Recently Tognneto et al. used a profilometric technique in different commercial IOLs. From the raw profile, the best-fit circle was subtracted to find differences between IOLs designs^12,13^. However, to limit the adjust of the raw profile to best-fit circle can lead to some limitations in the understanding of the IOLs because most of new designs are based on more complex surfaces.

The aim of this work is to test the feasibility of using profilometers to extract information about IOL surfaces design that allows a best understanding of optical IOL characteristics. A new purpose of best-fit surface will be introduced to achieve this goal. Best-fit circle and aspheric profile will be considered. Unlike to the articles published to date, in this work the two surfaces of the lens will be analyzed. This new adjustment method will allow to verify if the surfaces used in lens manufacturing are spherical, aspherical or high order aspherical. Finally, the profile study of a new trifocal IOL was included in this study.

## Material and methods

### Intraocular lenses

Four IOLs were used in this study, a standard monofocal IOL (Tecnis 1), a monofocal IOL that provides some depth of focus (Eyhance), a depth of focus IOL based on refractive optics (Mini Well) and a trifocal IOL based on diffractive optics (Synergy).

Tecnis 1 ZCB00 IOL (Johnson & Johnson Vision, Inc), is a monofocal biconvex IOL with spherical posterior surface and an anterior aspheric surface that provided a negative SA of − 0.27 μm in an optical zone of 6 mm ^12,14,15^.

Tecnis Eyhance ICB00 IOL, (Johnson & Johnson Vision, Inc), is a biconvex IOL with a spherical posterior surface and a high order aspheric anterior surface. As Tecnis 1 provided a negative SA of − 0.27 μm for 6 mm optical size to compensate corneal spherical aberration. The Tecnis Eyhance IOL is designed to provide distance vision comparable to a monofocal IOL, but the lens extends the depth of focus improving intermediate vision^12,14^.

The Mini WELL IOL (SIFI, Lavinaio, Italy) is a single-piece progressive Extended Depth of Focus (EDOF) IOL. The optic is based in wavefront surface design, and it is divided into three different annular zones. The inner and middle zones have different spherical aberrations with opposite signs, whereas the outer one is a monofocal aspherical zone^5,16^.

TECNIS Synergy IOL (Johnson & Johnson Vision, Inc), is a new propose for presbyopia correction that combining an EDOF and multifocal diffractive profile tries to provide continuous range of vision from far through near. The biconvex optic has a wavefront-designed aspheric anterior surface and a diffractive posterior optic^17,18^.

### Measurement method

The surface topography of the IOLs was measured by using a multimode optical profilometer (Zeta Instruments, model Z 300). The three-dimensional (3D) image of the surface along the diameter of the lens was obtained by confocal grid structured illumination, with a 100X objective. A stack of 400 images was acquired, covering a vertical range of 280 μm around a starting focal distance of the objective, previously set by autofocus. The field of view of a single image was 0.131 × 0.098 mm. The lens was held in a high-precision XY motorized platform which allows to automatize the acquisition of topographic maps across the diameter of the lens. Map stitching was performed by the software of the profilometer to display a compound 2D profile representative of a surface of the IOL. A set of 50 maps was needed to complete the diameter.

### Best-fit surface calculation process

**Figure 1:**
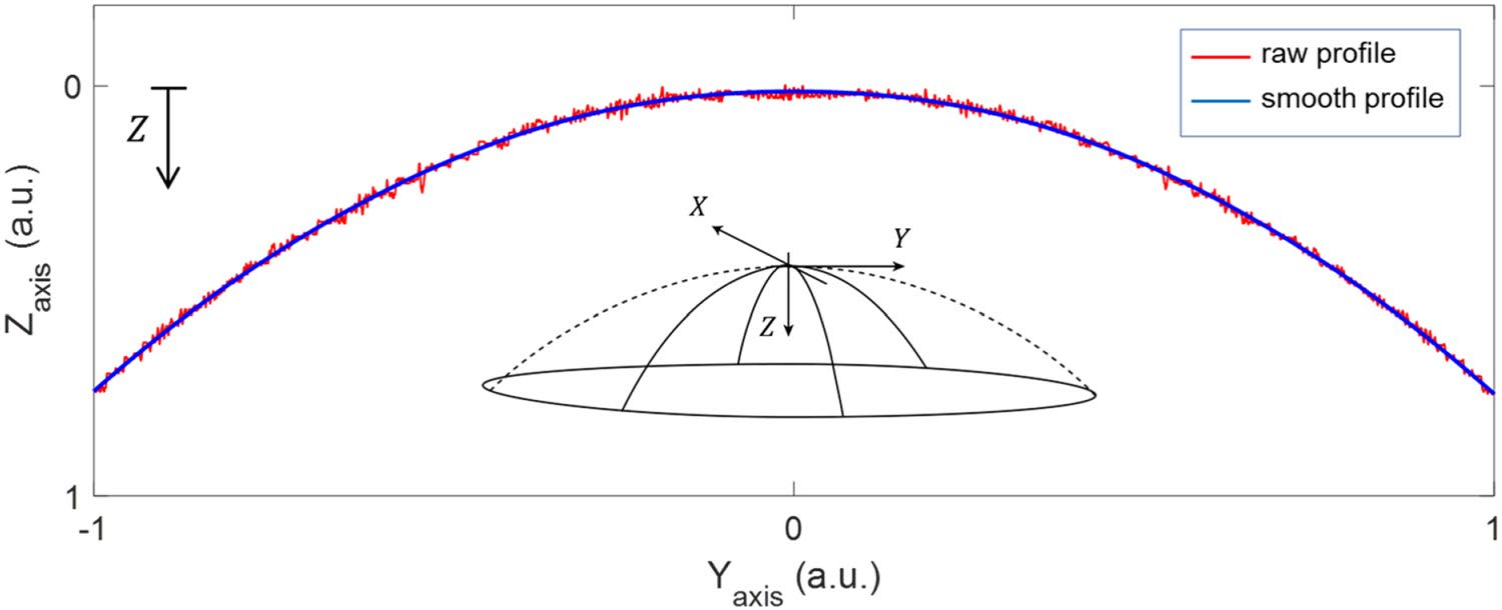
Example of a schematic raw profile and the corresponding smoothed profile obtained for an intraocular lens surface. Axes expressed in arbitrary units (a.u.).

The raw data extracted from the profilometer contained information on surface roughness as well as noise and possible artifacts generated during the measurement process. To figure out the geometrical shape of the surface, these non-desired effects had to be eliminated or reduced drastically. For this purpose, a smoothing process was performed by applying a least square weighted linear regression to the raw data. A second-degree polynomial model was used for the regression. The Fig. 1 showed, in a somewhat exaggerated example, the result of this process. The raw profile along the diameter of the IOL is represented, where Y-axis corresponds to the diameter dimension and Z-axis the height regarding a reference plane (sagitta).

### Surface description

A rotationally invariant optical surface can be described one-dimensionally by the surface sagitta value in the z direction as a function of the surface height ($h=\sqrt{{x}^{2}+{y}^{2}}$ ) by a combination of two terms^19^:

1 $$z\left(h\right)=\frac{{h}^{2}\rho }{1+\sqrt{1-\left(1+q\right)\left(h\rho \right)}}+f(h)$$

First term corresponds to the basic conical surface that is described by its curvature ρ as the reciprocal of the radius of curvature r_0_ and a conic constant (or asphericity) q. For q = 0, the basis is a spherical surface, and for q ≠ 0 the surfaces are considered aspherical. The basis term is followed by a series expansion as a function of the surface height *f(h).* Aspheric surface of higher order was defined when this function is necessary to be added to the basic conic surface^19^.

**Figure 2:**
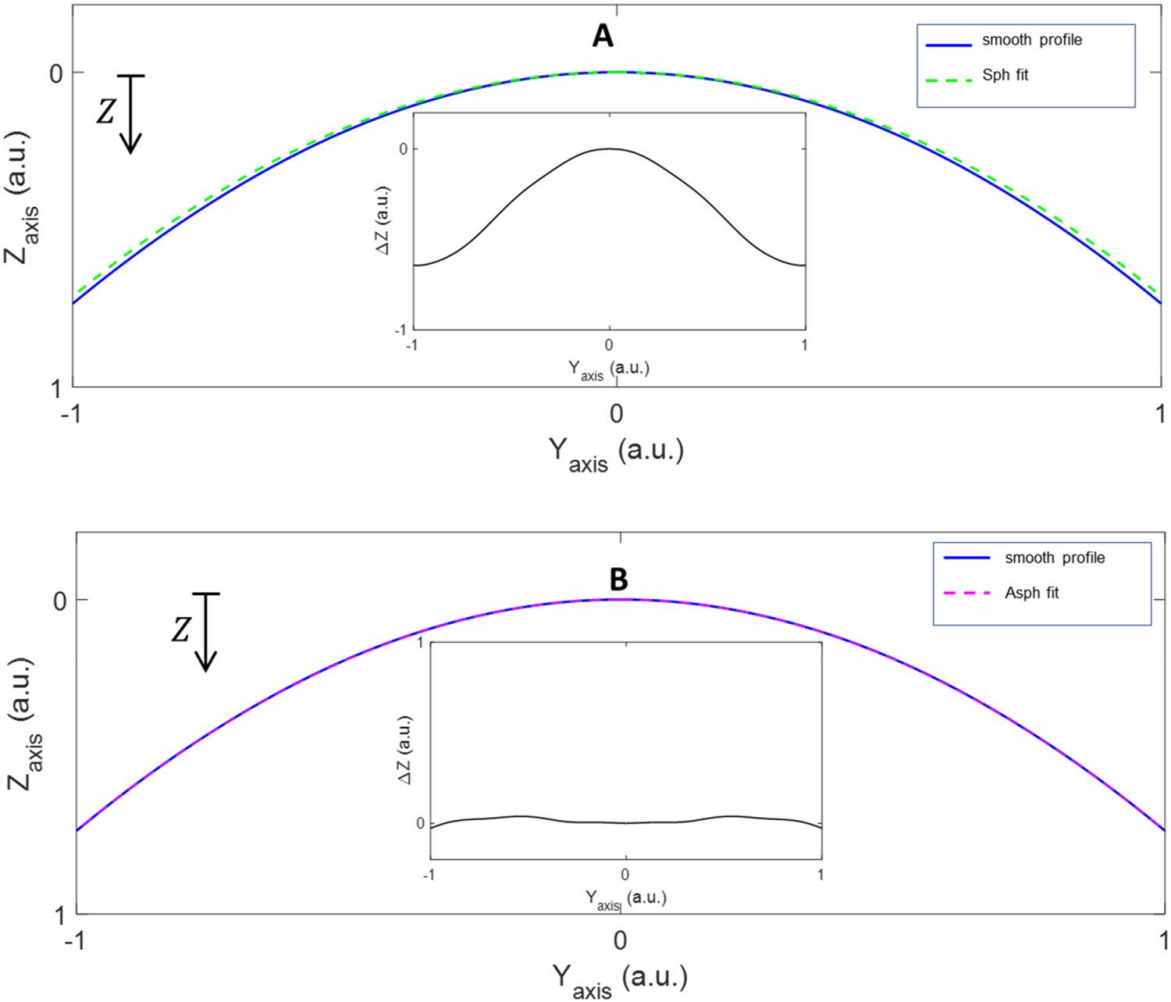
Example of a smoothed curve fitting to a sphere (**A**) or a sphere (**B**). Inserted in each figure is shown the differences of *Δz* regarding the reference surface.

Once roughness, noise, and artifacts were minimized, reliable information about the surface geometry could be extracted. With this objective and employing a nonlinear least-square curve fitting algorithm, the smoothed data were fitted to two kinds of functions: the sagitta of a spherical surface (*z*_*sph*_[ρ,0]) and the sagitta of an aspheric surface (*z*_*asph*_[ρ,q]), with one best fitting parameter, the curvature. To know the goodness of the fit, the difference (*Δz*) between *z*_*sphfit*_(h)[ρ_fit_,0] or *z*_*asphfit*_(h)[ρ_fit_,q_fit_] regarding the smooth profile in z-axis *z*_*smooth*_(h) [ρ,q] were obtained. This analysis revealed whether the smooth profile was spherical, aspheric or incorporated higher order terms. Figure 2 showed an example where it was evident that the surface fitted better to an aspheric surface (Fig. 2B) than a spherical one (Fig. 2A) since *Δz* = *z*_*asphfit*_[ρ,q]- *z*_*smooth*_ ≈0. An aspheric surface of higher order was considered when differences *Δz* were large considering both *z*_*sphfit*_ and *z*_*asphfit*_. In these cases, the expansion term *f(h)* would be necessary for the fit according to Eq. (1).

Both smoothing and fitting were performed using routines and algorithms written in Matlab (MATLAB, The MathWorks, Natick, MA).

## Results

### Raw profiles

**Figure 3:**
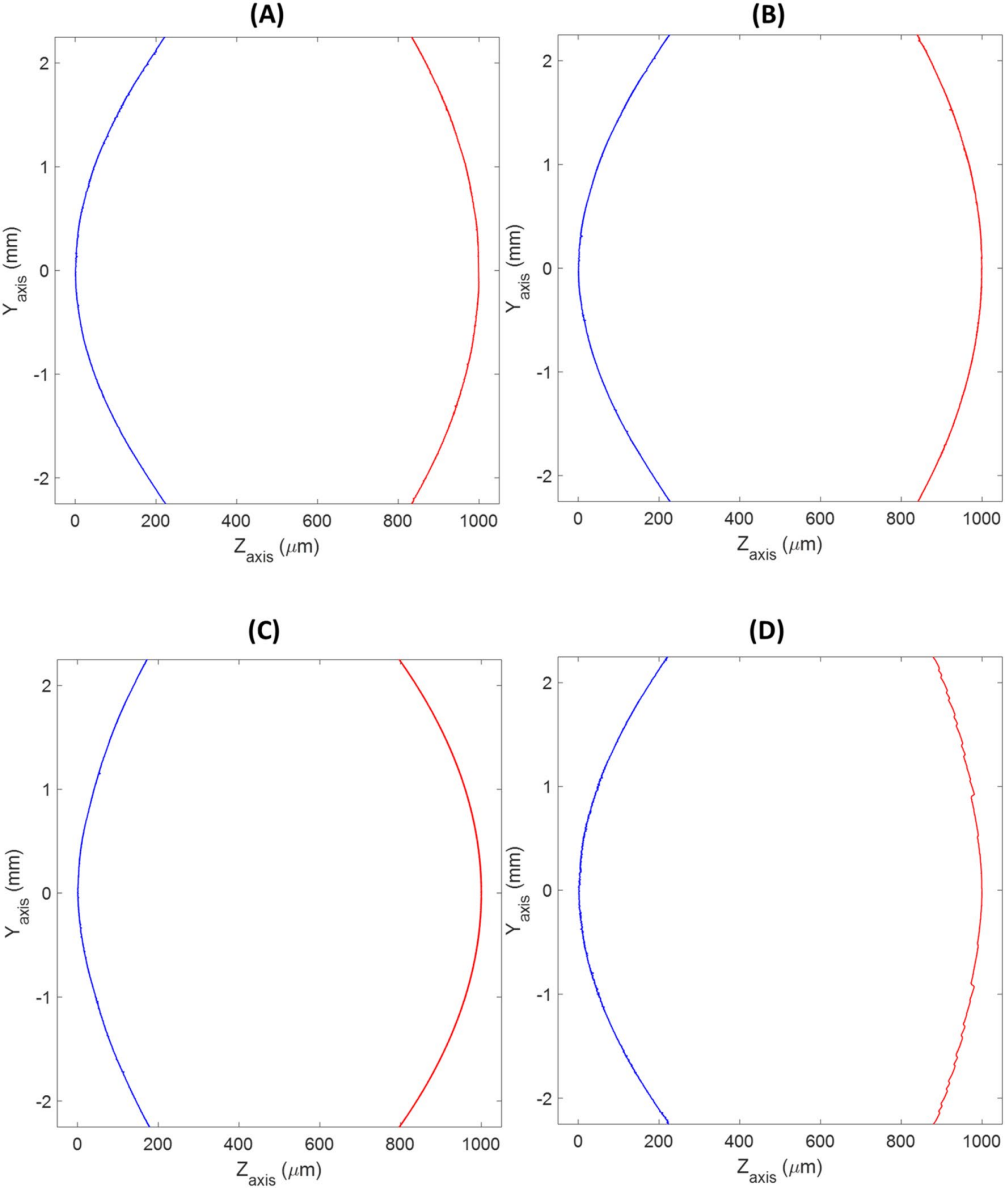
The 2D representations show the central raw profiles of first surface (blue) and second surface (red) of the LIOs. (**A**) Tecnis 1, (**B**) Eyhance, (**C**) Mini Well Ready and (**D**) Synergy.

Figure 3 showed the raw profiles extracted from the multimode optical profilometer. As seen, with this type of representation was very difficult to draw differences or conclusions. All surfaces (except the second surface of the Synergy IOL (Fig. 3D) that showed the typical diffractive echelette design) presented very similar shapes. Hence, further analysis is required to find out the real differences between the surfaces that can explain the different optical behaviours of each IOLs.

### Best-fit surface profiles

From smoothed profile, two options of best-fit surface were proposed: a sphere or asphere surfaces (see Eq. 1). Graphs derived from this analysis represented the difference (*Δz*) between heights of the best-fit surface and the smooth profile along the IOLs diameter (Y-axis).

**Figure 4:**
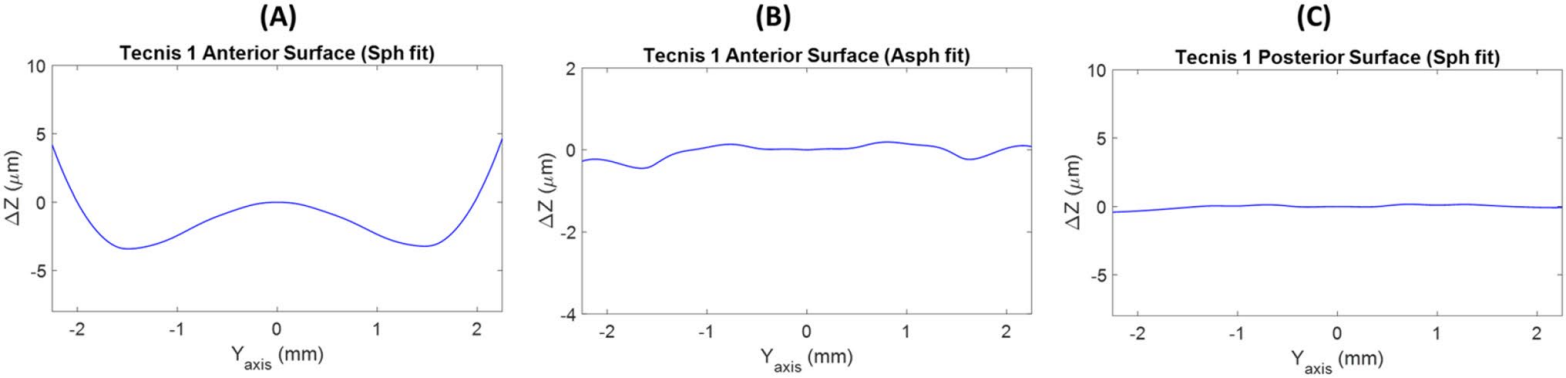
Subtraction lines obtained after the removal of the best-fit sphere (**A**) or asphere (**B**) of the anterior surface and the best-fit sphere of posterior surface (**C**) from the smooth profiles of the Tecnis 1 IOL.

As seen in Fig. 4, posterior surface of Tecnis 1 IOL was spherical. It showed a flat subtracting line (*Δz*≈0) after removal of the best-fit circle from the smooth profile (Fig. 4C). Regarding the anterior surface, differences of *Δz* up to 7 µm were obtained when a spherical surface reference was considered (Fig. 4A), while only 0.5 µm were observed using an aspherical surface reference (Fig. 4B). This result is compatible with considering the anterior surfaces as aspherical.

**Figure 5:**
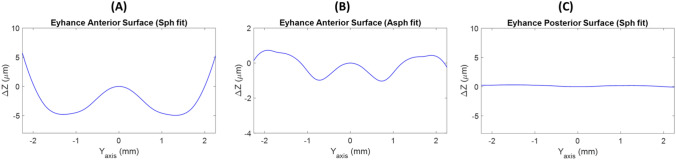
Subtraction lines obtained after the removal of the best-fit sphere (**A**) or asphere (**B**) of the anterior surface and best-fit sphere (**C**) of posterior surface from the smooth profiles of the Eyhance IOL.

In the Eyhance IOL, posterior surface was spherical (Fig. 5C) but anterior surface did not match any of our reference surfaces indicating a high order aspherical surface design (see Figs. 5A and B). In the anterior surface of the Eyhance, differences of *Δz* up to 10 µm were achieved when a spherical reference surface was fitted and up to 1 µm when the reference was an aspherical surface.

**Figure 6:**
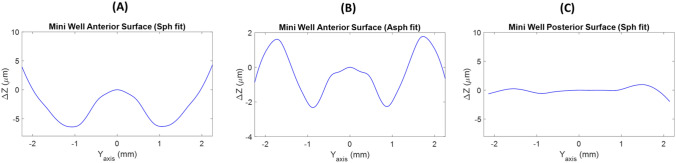
Subtraction lines obtained after the removal of the best-fit sphere (**A**) or asphere (**B**) of the anterior surface and best-fit sphere (**C**) of posterior surface from the smooth profiles of the Mini Well Ready IOL.

In the Mini Well Ready IOL, a best-fit sphere surface was obtained for the second surface (Fig. 6 C). However, neither a best-fit sphere surface nor a best-fit asphere surface were found for the anterior surface indicating a high order aspherical surface design. As seen in Figs. 6A and B, there were differences of *Δz* up to 8 µm and up to 3 µm when a best-fit sphere or asphere surfaces were respectively considered.

**Figure 7:**
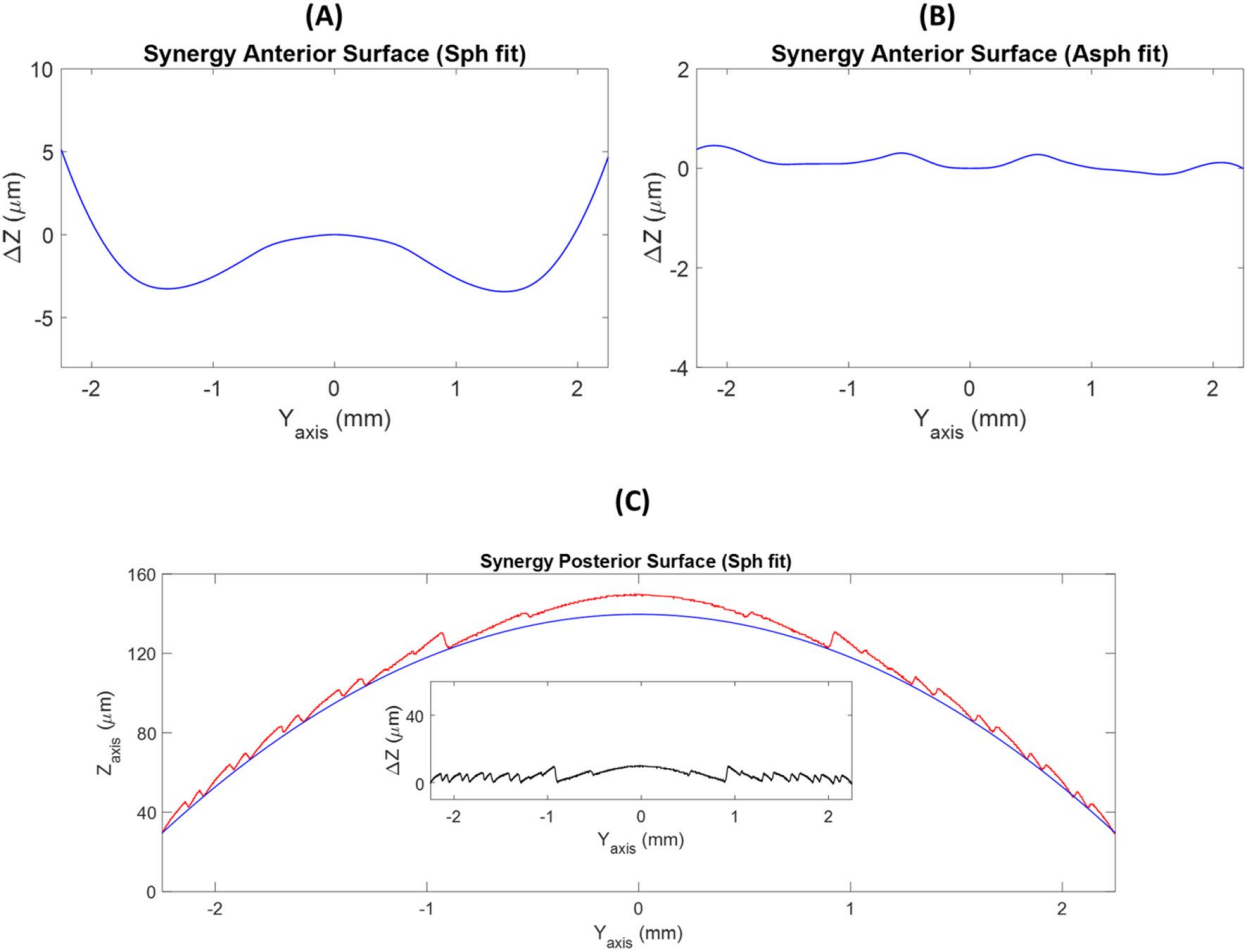
Subtraction lines obtained after the removal of the best-fit sphere (**A**) or asphere (**B**) from the smooth profiles of the anterior surface of the Synergy IOL. (**C**) Diffractive design of second surface and best-fit sphere for the echelette profile of the second surface of the Synergy IOL. The inset showed differences between complete profile and base curve fitted.

The anterior surface of the Synergy IOL was aspherical because up to 8 µm of difference were found when a best-fit sphere was considered (Figs. 7A and B). The second surface showed typical diffractive IOL surface (Fig. 7C). To obtain the refractive base curve on which the diffractive steps are sculpted, it was necessary to perform a further step in the analysis. Figure 7C showed two different diffractive patterns in each period of the diffractive step ^1^. Taking as reference the final point of each diffractive step, the base curve fitted to a spherical surface (*Δz≈*0). The inset of the Fig. 7C showed the difference between the echelette profile and the spherical curve fitted.

## Discussion

The study of the surface profile of intraocular lenses can provide valuable knowledge about the design and optical properties. Profilometers provide a raw profile that is necessary to smooth in order to fit to some known surface. To our knowledge, only studies using spheres as reference surfaces had been carried out. However, this assumption is insufficient for the analysis of the profiles of many IOLs on the market if optical characteristics want to be extracted.

It is well known that spherical surfaces provide positive spherical aberration (Z_40_) and with an asphere surface design, the primary spherical aberration can be controlled but not the higher spherical Zernike components^20,21^. Production of higher Zernike spherical orders (secondary, tertiary, etc.) generated from high order aspherical surfaces could providing different optical properties as higher depth of focus^5^. In this study an aspheric surface as a possible best-fit surface was proposed to ascertain whether IOL surface designs are spherical, aspherical, or higher-order aspherical. As consequence of this new approach, this study confirmed that the anterior surface of the Tecnis 1 IOL was aspherical and the second one was spherical as several reports have stated^12,14,15^ but not showed (see Fig. 4A–C).

Our method was able to demonstrate that posterior surface of the Eyhance IOL was spherical and the second surface did not match to a spherical or aspherical surface. According to Fidel et al. the wavefront aberration of Eyhance IOL provided by the second surface, had contributions of positive 6th-order and negative 8th-order of spherical aberration^14^. Therefore, differences observed in Fig. 5A and B of the *Δz* higher than 1 micron indicated a high order aspheric anterior surface design. Similar results were obtained for the Mini Well Ready IOL (see Fig. 6A and B). Second surface was spherical (Fig. 6C) and the anterior surface turned out high order aspheric surface design with differences of *Δz* higher than 3 microns. The Mini Well Ready IOL applies different surface design along the IOL surface providing positive and negative spherical aberrations in different zones of the lens^5,6,16^. In addition, as Camps et al. reported, Mini Well Ready provided spherical aberration of 4th and 6th order of opposite sign^5^. This high order aspheric surface design provides more depth of focus than the Eyhance as several authors reported by ^5,22–25^. Differences of *Δz* between Eyehance and Mini Well Ready IOLs when an aspherical surface reference could explain the light propagation differences between both IOLs.

Regarding the Synergy IOL, the anterior surface was aspherical (see Fig. 7B). However, the analysis of the first surface was more complicate due to the echelette design. Anyway, based on the knowledge of typical trifocal designs, it is possible to deduce that the base curve of the diffractive surface is a spherical surface (see Fig. 7C). This is the first time that this statement is reported.

The results of this study confirmed that the new possibility of fitting an aspherical surface as reference allows a more accurate deduction of the design of the lens surfaces and its optical properties. It is possible to know not only whether the design is spherical or aspherical but also whether it is high order aspherical. Furthermore, the high order aspherical surfaces provides spherical Zernike terms higher than 4^th^ order contributing to depth of focus generation. This method has been also useful to deduce the base curve used in diffractive surfaces as the Synergy IOL. Furthermore, the interpretation of these results can help to understand the optical behaviour of the IOLs. For instance, despite both Eyehance and Mini Well Ready are considered EDOF IOLs, our results showed high differences of *Δz* (when an aspheric fit surface was considered) for the Mini Well Ready IOL than for the Eyehance IOL. This result could be related to the generation of largest number of focus and more depth of focus in the Mini Well Ready IOL. In fact, Eyehance is considered as monofocal IOL with some depth of focus around the far focus ^14^, meanwhile, Mini Well Ready is considered as bifocal IOL with extended depth of focus around near and far focus^26^. Likewise, Synergy IOL, as our Fig. 7C showed, used at least two diffractive patterns in each period compatible with a trifocal diffractive IOL design^1^.

In conclusion, better knowledge of the design of intraocular lens surfaces will contribute to a better understanding of the optical and visual effects they can provide. These results can be used to implement and complete the simulations based on wavefront measurement and provide more information about the light pathway of IOLs.

## Data Availability

The datasets used and/or analysed during the current study are not available because the IOLs are protected by patent.
